# Sucrose Consumption Alters Serotonin/Glutamate Co-localisation Within the Prefrontal Cortex and Hippocampus of Mice

**DOI:** 10.3389/fnmol.2021.678267

**Published:** 2021-06-28

**Authors:** Kate Beecher, Joshua Wang, Angela Jacques, Nicholas Chaaya, Fatemeh Chehrehasa, Arnauld Belmer, Selena E. Bartlett

**Affiliations:** ^1^Addiction Neuroscience and Obesity Laboratory, Faculty of Health, School of Clinical Sciences, Translational Research Institute, Queensland University of Technology, Brisbane, QLD, Australia; ^2^Addiction Neuroscience and Obesity Laboratory, Faculty of Health, School of Biomedical Sciences, Translational Research Institute, Queensland University of Technology, Brisbane, QLD, Australia

**Keywords:** VGLUT3, 5-HT, pMAPK, serotonergic neuroplasticity, sucrose consumption, neurogenesis, microglia, addiction

## Abstract

The overconsumption of sugar-sweetened food and beverages underpins the current rise in obesity rates. Sugar overconsumption induces maladaptive neuroplasticity to decrease dietary control. Although serotonin and glutamate co-localisation has been implicated in reward processing, it is still unknown how chronic sucrose consumption changes this transmission in regions associated with executive control over feeding—such as the prefrontal cortex (PFC) and dentate gyrus (DG) of the hippocampus. To address this, a total of 16 C57Bl6 mice received either 5% w/v sucrose or water as a control for 12 weeks using the Drinking-In-The-Dark paradigm (*n* = 8 mice per group). We then examined the effects of chronic sucrose consumption on the immunological distribution of serotonin (5-HT), vesicular glutamate transporter 3 (VGLUT3) and 5-HT^+^/VGLUT3^+^ co-localised axonal varicosities. Sucrose consumption over 12 weeks decreased the number of 5-HT^–^/VGLUT3^+^ and 5-HT^+^/VGLUT3^+^ varicosities within the PFC and DG. The number of 5-HT^+^/VGLUT3^–^ varicosities remained unchanged within the PFC but decreased in the DG following sucrose consumption. Given that serotonin mediates DG neurogenesis through microglial migration, the number of microglia within the DG was also assessed in both experimental groups. Sucrose consumption decreased the number of DG microglia. Although the DG and PFC are associated with executive control over rewarding activities and emotional memory formation, we did not detect a subsequent change in DG neurogenesis or anxiety-like behaviour or depressive-like behaviour. Overall, these findings suggest that the chronic consumption of sugar alters serotonergic neuroplasticity within neural circuits responsible for feeding control. Although these alterations alone were not sufficient to induce changes in neurogenesis or behaviour, it is proposed that the sucrose consumption may predispose individuals to these cognitive deficits which ultimately promote further sugar intake.

## Introduction

Obesity is an expanding global health issue driven by the overconsumption of high sugar foods (Jacques et al., 2019a). Sugar is an addictive substrate, as demonstrated by a plethora of historical neurobehavioral studies. For sugar to be deemed an addictive substance, the four criteria need to be validated: bingeing, withdrawal, craving and cross-sensitisation. Sugar has been shown to elicit all hallmark signs of addicted behaviours: bingeing, cross-sensitisation (Avena and Hoebel, 2003), tolerance and cravings (Avena et al., 2005). In order to decrease the prevalence of diet-induced obesity in society, it is crucial to understand how sugar elicits an addictive response.

We have previously reported that varenicline, an FDA-approved nicotinic acetylcholine receptor (nAChR) partial agonist significantly reduced long-term 5% binge-like sucrose consumption (Shariff et al., 2016). For the first time, chronic long-term 25% sucrose consumption has been recently shown to augment weigh gain, elicit abnormal hyperlocomotion, impair cognitive function and alter neurogenesis (Beecher et al., 2021). Only continuous access to 25% sucrose has been investigated in mice, therefore, considering the effect of varenicline on 5% sucrose on reducing sugar intake, we had to examine chronic restricted (DID) 5% sucrose consumption.

The addictive nature of sugar may be due to sugar-induced cognitive neuroplasticity in both the hippocampus (Kanoski and Davidson, 2011) and prefrontal cortex (PFC) (Reichelt, 2016). These areas are focally responsible for memory and executive function, respectively. Many studies have investigated the effects of cognition and sugar, however, there is variability in the literature in terms of interspecies used, concentration of sugar, the type of sugar used and the method of administration. Most of the high sucrose diet impairing spatial and recognition memory have been conducted using a rat model (Jurdak et al., 2008; Jurdak and Kanarek, 2009). There remains relatively little data available on sugar-induced cognitive deficits in mice yet alone lower concentrations of sucrose.

The 5-HT-producing neurons are located in the dorsal (DR) or median (MR) raphe nuclei in the brainstem and project to brain regions including the dentate gyrus (DG) of the hippocampus (Gras et al., 2002; Amilhon et al., 2010) and PFC (Herzog et al., 2004; Amilhon et al., 2010; Sakae et al., 2019). Serotonin signalling has been broadly implicated in the central regulation of diet-induced obesity. Activation of central serotonin receptors excites anorexigenic neurons (Sohn et al., 2011; Roepke et al., 2012), inhibits orexigenic neurons (Heisler et al., 2006). A variety of serotonin receptor subtypes such as 5-HT_1B_, 5HT_2B_, 5HT_2C_, 5-HT_4_, and 5-HT_6_ mediate weight loss and satiety (Walsh et al., 1994; Cowen et al., 1995; Sargent et al., 1997; Heisler et al., 2002, 2006; Conductier et al., 2005; Jean et al., 2007; Nonogaki et al., 2007; Smith et al., 2009; Banas et al., 2011). On the other hand, knockout models of central serotonin receptors increases feeding and weight gain (Tecott et al., 1995; Bouwknecht et al., 2001; Xu et al., 2008, 2010). There is also evidence to suggest that serotonin selectively modulates the hedonic aspect of feeding. A selective serotonin reuptake inhibitor blunts the standard appetite-related responses to highly palatable or aversive tastants (Mathes et al., 2013). Additionally, the 5-HT system has been implicated in emotional eating in humans, polymorphisms within serotonin transporter linked polymorphic region (5-HTTLPR) are associated with stress-induced eating susceptibility (Chen et al., 2015) and obesity in childhood (Miranda et al., 2017), adolescence (Sookoian et al., 2007) and adulthood (Fuemmeler et al., 2008). Together these data demonstrate a relationship between serotonin signalling within the brain, and diet-induced obesity. However, the specific serotonergic projections that influence this behaviour remain unknown. It is therefore likely that serotonin innervation within regions of the brain associated with sugar consumption, such as the PFC and DG, are at least partially responsible for sugar consumption. Hence, we predicted that chronic sugar consumption may induce a change in the serotonin innervation at the PFC and DG, which may influence the likelihood of further consumption.

Reward seeking behaviour is proposed to be modulated through the co-transmission of 5-HT neurons and glutamate through the action of vesicular glutamate transporter 3 (VGLUT3) (Liu et al., 2014; Qi et al., 2014; Wang et al., 2019). Given the essential role of the PFC in dietary restraint (Jurdak and Kanarek, 2009; Higgs et al., 2012) and the DG in memory storage, we hypothesise that sucrose consumption may alter 5-HT/VGLUT3 innervation in these regions leading to less cognitive control over reward related behaviours, such as further sucrose consumption. Therefore, in the present study, we examined the impact of chronic sucrose consumption on serotonin innervation within the PFC and DG. We found that mice that have chronically consumed sucrose have a decreased number of 5-HT and 5-HT/VGLUT3 varicosities within the PFC and DG. The DG was also found to have a decreased number of VGLUT3 varicosities within the DG, but not in the PFC. We also found that sucrose consumption decreased the number of microglia within the DG, and increased the number of pMAPK neurons in the PFC.

## Materials and Methods

### Animals

Sixteen C57BL/6 male mice (4 weeks old) were trained for the 16 weeks using the Drinking-In-The-Dark (DID) model of binge-like consumption, adapted to sugar (Rhodes et al., 2005; Crabbe et al., 2009, 2011; Thiele et al., 2014; Belmer et al., 2018a; Patkar et al., 2019; Beecher et al., 2021). Mice were given access to one bottle of 5% (w/v) sucrose for a 2 h period Monday to Thursday and a 4 h period on Friday with acidified-filtered water available at all other times. The sugar solution was presented in 50 ml plastic falcon tubes fitted with rubber stoppers and a 6.35 cm stainless-steel sipper tube with double ball bearings. Sugar containing tubes were weighed prior to and 2 h (Monday to Thursday) or 4 h (Friday) after presentation. Sucrose intake and weights across the study can be seen in Supplementary Figure 1. Mouse weights were measured daily to calculate the adjusted g/kg intake. All procedures were approved by The University of Queensland and The Queensland University of Technology Animal Ethics Committees under approval QUT/053/18 and complied with the policies and regulations regarding animal experimentation and other ethical matters, in accordance with the Queensland Government Animal Research Act 2001, associated Animal Care and Protection Regulations (2002 and 2008), as well as the Australian Code for the Care and Use of Animals for Scientific Purposes, 8th Edition (National Health and Medical Research Council, 2014).

### Behavioural Testing

**FIGURE 1:**
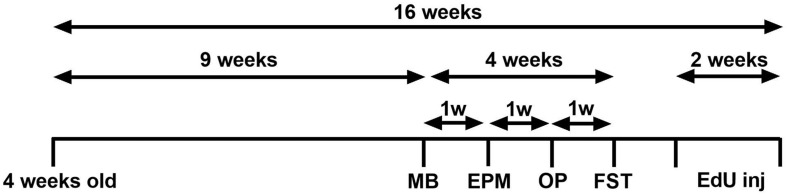
Experimental design of the study. Experimental design of the anxiety-related and depression-related marble burying (MB), elevated-plus-maze (EPM), open-field (OF), and forced swimming test (FST). Animals consumed 5% sucrose in the DID paradigm for 9 weeks prior to each behavioural testing and continued to be exposed to sucrose for a total of 16 weeks. Behavioural tests were conducted 1 week apart (across 4 weeks) after 24 h of sucrose withdrawal. After 14 weeks of sucrose consumption (1 week after behavioural tests concluded), three EdU injections were administered over 2 weeks (days 0, 7, 14). Animals were assigned into two groups: sucrose-withdrawn animals and water control.

Following 9 weeks of DID sucrose consumption, four behavioural tests were conducted to assess if the sucrose consumption model used produces emotional deficits. All behavioural tests have been performed extensively in our laboratory (Belmer et al., 2018a; Patkar et al., 2019; Beecher et al., 2021). Each test was conducted 1 week apart, over 4 weeks, see Figure 1.

For the testing of withdrawal-induced anxiety-like behaviour, experiments were carried out 24 h after the last drinking session of the week. Marble burying (MB) is used to test anxiety and obsessive-compulsive disorder. When mice are placed in the cage with marbles, they will bury marbles as a defence mechanism under conditions of stress. MB is performed in novel individual plastic cages (21 × 38 × 14 cm) containing 5 cm thick sawdust bedding. Ten glass marbles (diameter 10–12 mm) were arranged on the bedding evenly spaced in 2 rows of 5 marbles. After 20 min, the number of unburied marbles is averaged from counting by two experimenters blind to the treatments. A marble covered at least two-thirds (2/3) of its size by saw dust was considered as “buried” (Deacon, 2006; Belmer et al., 2018a; Patkar et al., 2019; Beecher et al., 2021).

Elevated-plus-maze (EPM) is another measure of anxiety based on the animal’s aversion to open spaces. EPM is conducted in an apparatus consisting of four arms (35 cm × 5 cm), elevated 50 cm above the floor. The closed arms are enclosed with 40 cm high walls. The experiment went for 5 min, with initial mouse placement in the centre, facing the open arm. The number of entries and time spent in each arm was recorded using ANY-maze tracking software (Stoelting, IL, United States) (Walf and Frye, 2007; Belmer et al., 2018a; Patkar et al., 2019; Beecher et al., 2021).

Open-field (OF) test is used to measure exploratory behaviour, general activity and anxious behaviour. This test is based on the animal’s aversion to bright light and open spaces. OF was performed in an open arena of 30 × 30 cm. The floor is divided into 16 equal squares (7 × 7 cm) and a central region of 10 × 10 cm is considered as the centre. Mice are initially placed in one corner, and allowed to explore freely for 10 min. The number of entries in the centre was recorded using the ANY-maze software (Bailey and Crawley, 2009; Belmer et al., 2018a; Beecher et al., 2021).

The forced swimming test (FST) is commonly used to access depressive-like behaviour and is used to test the efficacy of antidepressants. The test is based on the rodent’s response to the threat of drowning. The FST is conducted in a cylindrical glass container measuring 50 cm in height and a diameter of 20 cm. The immobility time was recorded using ANY-maze tracking software (Yankelevitch-Yahav et al., 2015; Belmer et al., 2018b; Beecher et al., 2021).

### Immunohistochemistry

Following all behavioural tests, a total of three injections of 5-ethynyl-2’-deoxyuridine (EdU; 50 mg/kg) were given over 2 weeks (days 0, 7, and 14) to all mice to label proliferative cells (Beecher et al., 2021). This dose has been reported to label all actively dividing precursors in the mouse subgranular zone of the DG (Mandyam et al., 2007). Twenty-four hours after the last EdU injection, mice were transcardially perfused with 4% paraformaldehyde. Brains were harvested and postfixed overnight at 4°C in 4% paraformaldehyde and then kept in 0.1 M phosphate buffer saline (PBS) containing 0.02% (w/v) sodium azide (PBS-azide) until histology and immunohistochemistry processing. Thirty micron-thick coronal vibratome sections were collected and kept floating in ice-cold PBS-azide. The sections were permeabilised in 1% Triton X100, 0.1% Tween-20 in PBS for 1 h and then incubated in blocking solution for 1 h at room temperature (2% normal goat serum-NGS, 0.3% Triton X100 and 0.05% Tween-20).

To assess if chronic sucrose consumption alters serotonin innervation, sections containing the PFC (Bregma +2.26 to +2.66) and DG of the hippocampus (Bregma –1.5 to –2.0 mm) were incubated with rat anti-5-HT (Millipore #MAB352, 1:100) for 48 h at room temperature followed by and guinea-pig anti-VGLUT3 (Synaptic System #135204), at 1:500 dilution (Belmer et al., 2019), and phosphor-p44/42 MAPK (Erk 1/2) (Thr 202/Tyr 204) (Jacques et al., 2019b) at 1:150, overnight at room temperature (#4370, Cell Signalling Technology, MA, United States). After three washes in the blocking solution, the slices were incubated for 4 h at room temperature with secondary antibodies diluted in the blocking solution: goat anti-rabbit-Alexa 488, goat anti-guinea pig-Alexa 647 (Thermo Fisher Scientific, #A11034 and #A21450, respectively, 1:500) and goat anti-rat biotinylated (Jackson Laboratory # 112-065-003, 1:200). For the biotinylated secondary antibody, sections were then incubated in streptavidin-CY3 for 30 min at room temperature.

Given the importance of serotonin in modulating adult hippocampal neurogenesis (Gould, 1999; Kolodziejczak et al., 2015), we analysed if chronic sucrose consumption would also alter neurogenesis. Sections for neurogenesis containing the hippocampus were incubated overnight at 4°C with primary antibodies: rabbit anti-DCX (Abcam #18723, 1:500); rabbit anti-GFAP (Dako, ZO334); mouse anti-Nestin (Millipore, MAB353); mouse anti-Calretinin (Millipore, MAB1568); mouse anti-Calbindin (Sigma-Aldrich, C9848); rabbit anti-NeuN (Millipore Sigma, ABN78); goat anti-Iba1 (Abcam, AB5076); rabbit anti-Olig 2 (Millipore, AB9610); and Edu Click-iT^TM^ EdU Alexa Fluor^TM^ 488 Imaging Kit (Thermo Fisher Scientific, C10637) (Beecher et al., 2021) and with corresponding secondary antibodies, for 2 h at room temperature: goat anti-mouse 594 (Thermo Fisher Scientific, A11032) and goat anti-rabbit 647 (Thermo Fisher Scientific, A27040). Sections were mounted in Prolong gold antifade mountant with DAPI (Thermo Fisher Scientific, P36934).

### Imaging and Analysis

Whole PFC and DG of the hippocampus from three coronal sections per animal group (*n* = 8) were imaged on the Olympus FV3000 confocal microscope using a 40 × oil-objective, ×1.5 numerical zoom and 0.5 z-step. Images were deconvolved using Huygens professional v16.10 (Scientific Volume Imaging, The Netherlands) with iteration number set at 100, quality threshold at 0.001, signal to noise ratio at 15 for the 4 channels and converted in. OIF for subsequent quantification for 5-HT, VGLUT3, 5-HT/VGLUT3, and pMAPK in Imaris 9.1.2 using Surface Reconstruction and Spot Detection functions as previously described (Belmer et al., 2017, 2019). Using surface rendering, fluorescence thresholding, and masking of unwanted immunolabelling, we obtained 3D objects (structures) of VGLUT3 labelling within 5-HT boutons. All images were batch processed using the same surface thresholding parameters and mean fluorescence intensities of VGLUT3 labelling within 5-HT boutons and image volumes were obtained from the surface statistics in Imaris.

Quantification of neurogenesis was counted on Neurolucida 360 (MBF Bioscience). Each neurogenesis stage (stage 1: EdU+/GFAP+/Nestin−; stage 2: EdU+/Nestin+/GFAP-; stage 3: EdU+/DCX+; stage 4: EdU+/calretinin+/NeuN+, and stage 5: EdU+/calbindin+/NeuN+) as well as microglia (IBA-1+) was counted by an experimenter blind to the treatment, averaged per animal and plot as mean ± SEM for each group (Belmer et al., 2018a; Patkar et al., 2019; Beecher et al., 2021). The density of counted cells will be normalised to the volume of granular cell layer sampled in each group.

### Statistics

Comparisons between groups were statistically analysed using *t*-tests through GraphPad Prism 9 (Graph Pad Software Co., CA, United States). A *p* < 0.05 will be considered significant. All values are expressed as the mean ± SEM.

## Results

### Sucrose Alters the Density of the Neuronal Plasticity Marker pMAPK in the PFC

**FIGURE 2:**
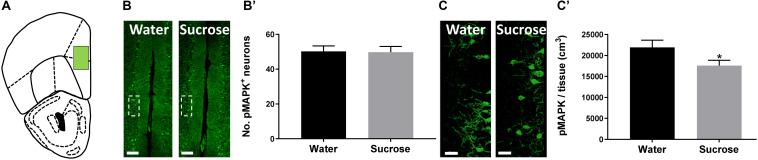
Neuroplastic changes after sucrose consumption. **(A)** Schematic drawing showing the location of the acquired micrographs (prefrontal cortex). **(B)** Low-magnification representative image of the prefrontal cortex showing the distribution of the pMAPK positive cells. Scale: 250 μm. **(B’)** Quantification of pMAPK positive neurons showed no difference after sucrose consumption. **(C)** High-resolution representative image of pMAPK somatodendritic staining reconstruction. Scale: 50 μm. **(C’)** The volume of pMAPK neurons/staining was reduced after sucrose consumption. Data are expressed as mean ± SEM (*t*-test). *p < 0.05, compared with vehicle (*n* = 8 per treatment group).

The PFC is known to exert restraint over hedonic eating behaviour (Hare et al., 2009) and a decrease in PFC activity has been associated with the breakdown of healthy dieting (Demos et al., 2011). Additionally, serotonin may also induce pMAPK-associated neuroplasticity (Michael et al., 1998). Therefore, we predicted that sucrose consumption will decrease plasticity within the PFC, reducing its inhibitory control over hedonic food consumption. To evaluate this, counted the number of pMAPK-immunoreactive neurons and reconstructed pMAPK neurons using Imaris to quantify the volume of pMAPK (neuroplasticity marker) neurons/staining in the PFC (Figure 2A). No change in the number of pMAPK neurons was observed in the PFC (Figures 2B,B’; *p* = 0.9177, *t*-test). A significant reduction in the volume of pMAPK neurons/staining in the PFC between sucrose and water consuming mice was detected (Figures 2C,C’; *p* = 0.0434, *t*-test). Although we have not seen a difference in the number of pMAPK neurons we have observed a reduction in the dendritic complexity of these neurons suggesting a decrease in the plasticity of these neurons.

### Sucrose Alters Serotonin Innervation in the PFC and DG

**FIGURE 3:**
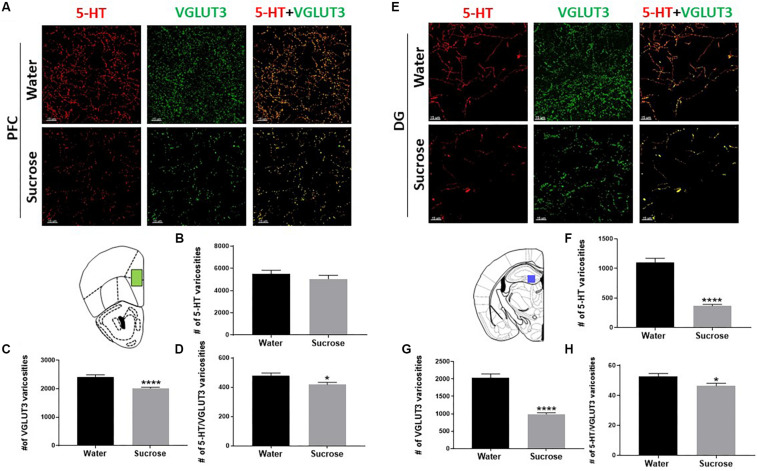
Sucrose alters serotonin innervation in the PFC and DG of the hippocampus. A high-resolution representative image of the PFC **(A)** and DG of the hippocampus **(E)** showing the distribution of the reconstructed 5-HT, VGLUT3, and 5-HT/VGLUT3 co-localisation surfaces between water and sucrose consuming animals (Scale: 50 μm). Sucrose drinking does not alter the density of 5-HT varicosities **(B)** however, decreases the density of VGLUT3 varicosities **(C)** and the density of 5-HT/VGLUT3 co-localised varicosities **(D)** in the PFC. Sucrose drinking selectively decreases the density of varicosities of 5-HT **(F)**, density of VGLUT3 varicosities **(G)** and density of 5-HT/VGLUT3 **(H)** co-localised varicosities in the DG of the hippocampus. Data are presented as mean ± S.E.M (*t*-test); *n* = 8 mice/group. *p < 0.05, ****p < 0.0001. PFC, prefrontal cortex; DG, dentate gyrus.

Co-release of serotonin and glutamate in the limbic system has been proposed to play a pivotal role in the development of emotional deficits like anxiety, depression and addiction. As the serotonin system is widely implicated in ethanol and sucrose consumption, and VGLUT3 expression is modulated by alcohol and sugar intake (Tukey et al., 2013; Vrettou et al., 2019), we investigated the density of varicosities that would be able to release and co-release 5-HT and glutamate in the PFC (Figure 3A) and DG (Figure 3E). Sucrose drinking selectively reduces the number of 5-HT varicosities in the DG (Figure 3F; ^****^p = 0.0001, *t*-test) not in the PFC (Figure 3B; NS, *p* = 0.3394, *t*-test). Sucrose drinking also selectively reduces the density varicosities that release VGLUT3 in PFC (Figure 3C; ^****^p = 0.0001, *t*-test) and DG (Figure 3G; ^****^p = 0.0001, *t*-test). Sucrose drinking selectively decreased the number of putative 5-HT/VGLUT3 co-release sites in both the PFC and DG (Figures 3D,H; D: ^∗^*p* = 0.0201, H: ^∗^*p* = 0.0238, *t*-test). Here for the first time, we have shown chronic restricted sucrose consumption produces changes in the serotonergic innervation of the PFC and DG of the hippocampus.

### Sucrose Does Not Alter Adult Hippocampal Neurogenesis but Reduces DG Microglia Density

It has well been established that a reduction in serotonin levels is correlated with anxiety and depression. This depression and anxiety are then partially caused by the impairment of neurogenesis which is known as the neurogenic theory of depression and anxiety (Miller and Hen, 2015). Serotonin may therefore also control sucrose intake through cognitive changes resulting from neurogenesis within the DG. Fructose-consuming rats (van der Borght et al., 2011) and mice (Cisternas et al., 2015) show neurogenic deficits within the DG. The importance of serotonin in neurogenesis has been shown as the selective serotonin reuptake inhibitors mediate their anti-depressive responses through binding 5-HT_2B_ receptors in the DG (Diaz et al., 2012) and subsequently altering hippocampal neurogenesis (Alenina and Klempin, 2015). Upon 5-HT_2B_ receptor activation, DG microglia migrate toward neuroblast cells (Kolodziejczak et al., 2015), a process essential for neurogenesis (Kreisel et al., 2019). As we have observed a reduction in density of 5-HT, VGLUT3 and 5-HT/VGLUT3 varicosities, we therefore assessed sucrose effect on emotional behaviour after 9 weeks of sucrose, and neurogenesis after 16 weeks of sucrose consumption.

**FIGURE 4:**
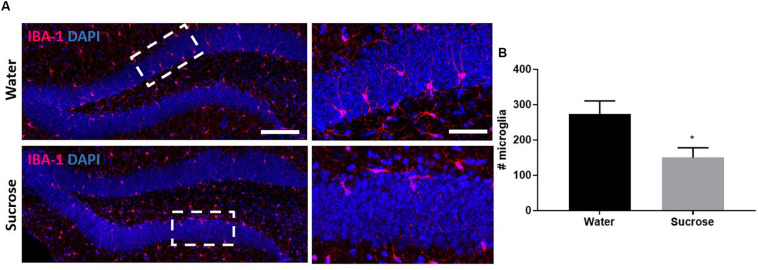
Sucrose consumption alters microglia in the dentate gyrus of the hippocampus. **(A)** Representative images at both 4× (left images; scale bar = 100 μm) and 40× magnification (field indicated by white boxes on 4× magnification images; right images; scale bar = 10 μm). **(B)** Long-term sucrose consumption reduces the number of microglia (magenta) in the DG of the hippocampus. All images are co-localised with DAPI (blue). Data are presented as mean ± S.E.M (*t*-test); *n* = 8 mice/group. *p < 0.05.

We performed four behavioural tests assessing anxiety and depression: MB, EPM, OF and FST. Surprisingly, we did not observe any anxiety-like behaviour or depressive-like symptoms after sucrose withdrawal (Supplementary Figure 2). Supporting the neurogenic theory of depression, we also did not observe any neurogenic deficits across any stage of neurogenesis in the DG of the hippocampus (Supplementary Figure 3) which was interesting after observing changes in serotonin and knowing that serotonin mediates DG neurogenesis. Glial cells are key mediators throughout neurogenesis, therefore we also assessed glial cells after sucrose consumption. We did not see any changes in number of oligodendrocyte progenitor cells; however, we did observe a reduction in the number of microglia in the DG as a result of sucrose consumption (Figures 4A,B; ^∗^*p* = 0.0243). The dual impact of decreased 5-HT and decreased microglia was not sufficient to elicit any negative impact on neurogenesis.

## Discussion

**FIGURE 5:**
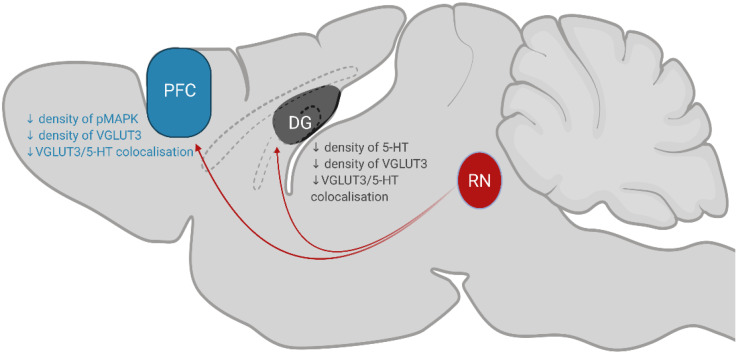
Summary of key findings. Serotonergic neurons in the RN (coloured red; dorsal, median and caudal raphe nuclei) project to the PFC and DG to modulate behaviours associated with eating. Chronic, low-concentration sucrose consumption causes a decrease in the density of pMAPK, VGLUT3 and 5-HT/VGLUT3 co-localisation in the PFC (coloured blue). In the DG of the hippocampus (coloured grey), sucrose consumption decreases the density of 5-HT, VGLUT3 and 5-HT/VGLUT3 co-localisation. RN, raphe nuclei; DG, dentate gyrus; PFC, prefrontal cortex.

This study demonstrates that chronic consumption of low concentration of sucrose leads to a decrease in the expression of the plasticity marker pMAPK and reduction in serotonin innervation within the PFC and DG, please see Figure 5 for summary of key findings. Given that these structures modulate emotions and behaviours associated with feeding (Kanoski and Davidson, 2011; Reichelt, 2016), these findings demonstrate how chronic sugar consumption can alter the central regulation of feeding from a molecular perspective. It is interesting that we do not observe any behavioural changes considering the effect of varenicline on reducing 5% sucrose intake (Shariff et al., 2016). However, small changes in serotonin co-localisation, neuroplasticity and microglia were observed. These results may predispose individuals to the delirious effects seen at higher concentrations of sucrose which ultimately promote further sugar intake.

Both high sugar and high-fat diets reduce the expression of genes involved in neuroplasticity. High-fat diets have shown to reduce hippocampal levels of brain-derived neurotrophic factor (BDNF) as well as cyclic AMP-response element-binding protein (CREB) mRNA and protein levels (Maniam et al., 2016). BDNF controls CREB activation and is important in memory formation with pMAPK being downstream of BDNF and upstream of CREB. Greater activation of prefrontal pMAPK has been observed following binge-ethanol consumption (Agoglia et al., 2015). Additionally, serotonin (Michael et al., 1998) and serotonin receptors (Yuen et al., 2008; Cui et al., 2016) also modulate pMAPK-associated neuroplasticity. Therefore, we wanted to determine the effect of sucrose consumption on pMAPK expression. In contrast to ethanol exposure (Agoglia et al., 2015), we observed a reduction in the overall immunoreactivity of pMAPK in the PFC. Changes in dendritic MAPK phosphorylation at the PFC, similar to what we have reported, has been correlated with altered apical dendritic structure (Papadeas et al., 2008). Therefore, dendritic mapping of the PFC following chronic sucrose consumption could be conducted to confirm if these structural changes are also present. In summary, these results suggest that sucrose negatively affects neuroplasticity in the PFC. Future work can investigate these regions associated with cognitive function by performing behavioural testing such as the Morris Water Maze.

The co-release of 5-HT and glutamate has been evidenced by the presence of the vesicular glutamate transporter 3 (VGLUT3) in some 5-HT varicosities and the co-release of the 2 neurotransmitters has been proposed to play an important role in controlling the reward system (Liu et al., 2014; Sengupta et al., 2017) and anxiety-related behaviours (Amilhon et al., 2010; Sakae et al., 2019). We therefore assessed the effect of sucrose consumption on the distribution of co-localised serotonin/glutamate in the PFC and DG. We observed a reduction in the density of 5-HT varicosities only in the DG, not the PFC. However, we saw a reduction in the density of VGLUT3 boutons in both DG and PFC and a reduction in the density of 5-HT/VGLUT3 varicosities. Decreased VGLUT3 expression may be associated with susceptibility to stress (Zou et al., 2020), however, the role VGLUT3 plays in sucrose consumption is unknown.

Cognitive and behavioural changes following VGLUT3 knockout/downregulation may not be regulated by serotonergic neurons. Neuronal projections from the dorsal raphe that contain VGLUT3, but not 5-HT have been previously documented (Commons, 2009; Hioki et al., 2010). These non-serotonergic neurons have also been implicated in reward (McDevitt et al., 2014) and have even been shown to mimic addictive phenotypes produced by VGLUT3 knockout. For example, the knockout of specifically cholinergic, but not serotonergic VGLUT3 neurons, is responsible for the aforementioned amphetamine-sensitisation (Mansouri-Guilani et al., 2019). It is therefore important that future studies be performed which immunologically dissect these VGLUT3 projections, for example by additionally probing for acetylcholine axons.

It is interesting that we did not see a reduction in 5-HT in the PFC yet we saw reduction in VGLUT3 and 5-HT/VGLUT3 varicosities considering the proposed mechanism of VGLUT3 is to synergise the filling of 5-HT and glutamate within synaptic vesicles (Amilhon et al., 2010). In an assessment of multiple brain regions, only the hippocampus displayed decreased serotonin levels as a result of VGLUT3 knockout (Amilhon et al., 2010). This suggests there is likely a unique mechanism within the hippocampus in which VGLUT3 modulates 5-HT uptake. Therefore, the decrease in DG 5-HT varicosities seen in sugar consuming mice may be a downstream effect of reduced VGLUT3 varicosities at the DG. As pharmacological agents that specifically target VGLUT3 are still being developed (Poirel et al., 2020), it is difficult to directly manipulate VGLUT3 activity *in vivo* to test this hypothesis. Alterations of the serotonergic and glutamatergic co-localisation within PFC and DG are supported by immunohistochemistry data. The co-localised serotonergic and glutamatergic alterations observed within PFC and DG need to be confirmed using functional studies. Future studies can measure the concentration of serotonin or glutamate *in vivo* using microdialysis studies. Functional experiments such as *ex vivo* electrophysiological studies will validate whether serotonin and glutamate are released from the same axons.

We also report that the DG microglial population is decreased following sucrose consumption. Microglia phagocytose apoptotic neuronal precursors to promote hippocampal neurogenesis under physiological conditions (Sierra et al., 2010) through the secretion of a plethora of neurogenic factors (Battista et al., 2006; Butovsky et al., 2006). Microglial signalling in the adult DG has also been shown to alter addictive behaviour (Rivera et al., 2019). Our results show that sucrose consumption influences neurogenesis through two possible mechanisms: a decrease in the number of microglia, as well as a decrease in 5-HT. Given that 5-HT promotes microglial migration in the DG during neurogenesis (Kolodziejczak et al., 2015; Kreisel et al., 2019), we expected to detect a resulting decrease in neurogenesis. Similar rodent studies using a high-fat diet have recorded decreased neurogenesis in mice (Yoo et al., 2014) and unaltered neurogenesis in rats (Rivera et al., 2013).

Our results show that, despite decreases in 5-HT and microglia in the DG, neurogenesis was unaltered in sucrose consuming mice. Therefore, these mice may not have been exposed to sucrose for a length of time where the decreased serotonergic innervation in the DG to alter neurogenesis, given that to higher concentrations of sucrose for 12 weeks elicits neurogenic deficits (Beecher et al., 2021). For example, after 10 days of SSRI administration, the DG of Sprague-Dawley rats did not display altered neurogenesis, but did show increased neuroplasticity (Pawluski et al., 2020), suggesting the onset of plasticity in this region precedes alterations in neurogenesis. We therefore hypothesised that our mice were not consuming sucrose long enough for the altered DG serotonergic input to impact neurogenesis. This potential delay in neurogenesis also suggests that higher sucrose concentrations may increase the speed of neuroplastic events, given that similar studies with similar timeframes of consumption have used higher sucrose concentrations (Kim et al., 2018; Xu and Reichelt, 2018; Beecher et al., 2021). However, sucrose consumption may not decrease the overall amount of neurogenesis but may impact the quality of the generated circuits. For example, transient microglial ablation within the olfactory bulb (another site for adult neurogenesis), results in the same number of adult neurons (granular cells) being produced, however their synaptic connectivity and odour detection is dampened (Wallace et al., 2020). This may explain why no changes in neurogenesis were seen in this study despite decreased microglial presence and 5-HT innervation within the DG. A trend does exist suggesting that sucrose consumption may increase microgliogenesis. This trend may represent a compensatory mechanism to restore the depleted microglial population seen after sucrose consumption and could potentially contribute to the lack of behavioural change.

Surprisingly, no changes in behaviour were identified across the four tests conducted. This indicates that the changes in glutamate/serotonin co-release sites detected within the PFC, as well as decreased microglial cell count and 5-HT varicosities in the DG were not sufficient to produce emotional or cognitive deficits. Once again, this may be due to inadequate exposure to sucrose, given the delay needed to impact neurogenesis (Pawluski et al., 2020), as well as the relatively low concentration of sucrose used.

This study demonstrates the potency of chronic, low-concentration sucrose consumption in producing intricate alterations in serotonergic neuroplasticity within key brain regions associated with feeding. Although the findings of this study suggest that sucrose consumption does not alter adult hippocampal neurogenesis, we have previously shown that sucrose alters neurogenesis at higher concentrations of sucrose (Beecher et al., 2021). Therefore, it is suggested that higher sucrose concentrations, and increased exposure to the sucrose solution is required to detect more specific changes in neurogenesis. Additionally, while proliferation in the DG was assessed in response to a high-sucrose diet, cell death was not studied. High-fat diets in rodents cause increased DG apoptosis in rats (Maniam et al., 2016) as well as decreased DG neuroprogenitor cell survival in mice (Yoo et al., 2014). Therefore, DG cell death should be assessed in sucrose consuming mice through TUNEL assays in order to fully understand sucrose’s effect on neurogenesis. Lastly, the current study is limited to only male C57BL/6 mice. Differences in diet-responsive neurogenic defects exist between species (Rivera et al., 2013; Yoo et al., 2014) and sex-specific differences in serotonergic function have been documented within the rat PFC (Brivio et al., 2018). Strain-specific differences in VGLUT3 expression and knockout phenotype also exist (Sakae et al., 2019). Care should therefore be taken when extrapolating these results into contexts of female rodents, or other mouse strains. Despite these limitations, this study provides novel insights into the mechanisms of sugar-induced neuroplasticity, with potential implications for obesity and eating disorder research.

## Data Availability Statement

The original contributions presented in the study are included in the article/Supplementary Material, further inquiries can be directed to the corresponding author/s.

## Ethics Statement

All procedures were approved by The University of Queensland and The Queensland University of Technology Animal Ethics Committees under approval QUT/053/18 and complied with the policies and regulations regarding animal experimentation and other ethical matters, in accordance with the Queensland Government Animal Research Act 2001, associated Animal Care and Protection Regulations (2002 and 2008), as well as the Australian Code for the Care and Use of Animals for Scientific Purposes, 8th Edition (National Health and Medical Research Council, 2014).

## Author Contributions

KB, AB, and SEB were responsible for study concept and design. KB, JW, and AJ carried out drinking experiments and behavioral experiments. KB, AJ, and NC performed immunohistochemistry and imaging. KB, JW, and FC analyzed the data, interpreted the findings, and drafted the manuscript and figures. FC, AB, and SEB edited the manuscript.

## Conflict of Interest

The authors declare that the research was conducted in the absence of any commercial or financial relationships that could be construed as a potential conflict of interest.

## Supplementary Material

Supplementary Figure 1 Five percentage sucrose consumption and weight. **(A)** After 16 weeks of access to 25% sucrose, mice exhibited stable levels of sucrose intake around 3–6 g/kg/2 h (mean 4.4 g/kg/2 h indicated by the red line). **(B)** No weight change was observed across the 16 weeks. Data are presented as mean ± S.E.M; *n* = 8 mice/group.

Supplementary Figure 2 No change observed in anxious behaviour as seen by the number of marbles buried in marble burying test **(A)**, number of open arm entries in the elevated-plus-maze **(B)**, number of entries into the centre in the open-field test **(C)**, no change in depressive like behaviour as seen by immobility time in the forced swimming test **(D)**. Data are presented as mean ± S.E.M (*t*-test); *n* = 8 mice/group.

Supplementary Figure 3 **(A)** No change in the total number of EdU + cells, suggesting no change in overall proliferation. No change in proportion of EdU + co-localised with any stage of neurogenesis: stage 1: glial fibrillary acidic protein (GFAP, **B**); stage 2: Nestin **(C)**; stage 3: Doublecortin (DCX, **D**); stage 4: calretinin (CalR, **E**) and stage 5: calbindin (CalB, **F**) suggesting no change in neurogenesis. No change in proportion of EdU + co-localised with glial populations: oligodendrocyte (olig2, **G**) and microglia (IBA, **H**). Data are presented as mean ± S.E.M (*t*-test); *n* = 8 mice/group.

## References

[B1] Agoglia A. E. Sharko A. C. Psilos K. E. Holstein S. E. Reid G. T. Hodge C. W. (2015). Alcohol alters the activation of ERK1/2, a functional regulator of binge alcohol drinking in adult C57BL/6J mice. *Alcohol. Clin. Exp. Res.* 39 463–475. 10.1111/acer.12645 25703719 PMC4348173

[B2] Alenina N. Klempin F. (2015). The role of serotonin in adult hippocampal neurogenesis. *Behav. Brain Res.* 277 49–57. 10.1016/j.bbr.2014.07.038 25125239

[B3] Amilhon B. Lepicard È Renoir T. Mongeau R. Popa D. Poirel O. (2010). VGLUT3 (Vesicular Glutamate Transporter Type 3) contribution to the regulation of serotonergic transmission and anxiety. *J. Neurosci.* 30 2198–2210. 10.1523/JNEUROSCI.5196-09.2010 20147547 PMC6634029

[B4] Avena N. M. Hoebel B. G. (2003). A diet promoting sugar dependency causes behavioral cross-sensitization to a low dose of amphetamine. *Neuroscience* 122 17–20. 14596845 10.1016/s0306-4522(03)00502-5

[B5] Avena N. M. Long K. A. Hoebel B. G. (2005). Sugar-dependent rats show enhanced responding for sugar after abstinence: evidence of a sugar deprivation effect. *Physiol. Behav.* 84 359–362. 10.1016/j.physbeh.2004.12.016 15763572

[B6] Bailey K. R. Crawley J. N. (2009). “Anxiety-related behaviors in mice,” in *Methods of Behavior Analysis in Neuroscience*, 2nd Edn, ed. Buccafusco J. J. (Boca Raton, FL: CRC Press/Taylor & Francis). 21204329

[B7] Banas S. M. Doly S. Boutourlinsky K. Diaz S. L. Belmer A. Callebert J. (2011). Deconstructing antiobesity compound action: requirement of serotonin 5-HT 2B receptors for dexfenfluramine anorectic effects. *Neuropsychopharmacology* 36 423–433. 10.1038/npp.2010.173 20927048 PMC3055663

[B8] Battista D. Ferrari C. C. Gage F. H. Pitossi F. J. (2006). Neurogenic niche modulation by activated microglia: transforming growth factor beta increases neurogenesis in the adult dentate gyrus. *Eur. J. Neurosci.* 23 83–93. 10.1111/j.1460-9568.2005.04539.x 16420418

[B9] Beecher K. Alvarez Cooper I. Wang J. Walters S. B. Chehrehasa F. Bartlett S. E. (2021). Long-term overconsumption of sugar starting at adolescence produces persistent hyperactivity and neurocognitive deficits in adulthood. *Front. Neurosci.* 15:670430. 10.3389/fnins.2021.670430 PMC8215656 34163325

[B10] Belmer A. Beecher K. Jacques A. Patkar O. L. Sicherre F. Bartlett S. E. (2019). Axonal Non-segregation of the vesicular glutamate transporter VGLUT3 within serotonergic projections in the mouse forebrain. *Front. Cell. Neurosci.* 13:193. 10.3389/fncel.2019.00193 31133811 PMC6523995

[B11] Belmer A. Klenowski P. M. Patkar O. L. Bartlett S. E. (2017). Mapping the connectivity of serotonin transporter immunoreactive axons to excitatory and inhibitory neurochemical synapses in the mouse limbic brain. *Brain Struct. Funct.* 222 1297–1314. 10.1007/s00429-016-1278-x 27485750 PMC5368196

[B12] Belmer A. Patkar O. L. Lanoue V. Bartlett S. E. (2018a). 5-HT1A receptor-dependent modulation of emotional and neurogenic deficits elicited by prolonged consumption of alcohol. *Sci.c Rep.* 8:2099. 10.1038/s41598-018-20504-z 29391482 PMC5794771

[B13] Belmer A. Quentin E. Diaz S. L. Guiard B. P. Fernandez S. P. Doly S. (2018b). Positive regulation of raphe serotonin neurons by serotonin 2B receptors. *Neuropsychopharmacology* 43 1623–1632. 10.1038/s41386-018-0013-0 29453444 PMC5983540

[B14] Bouwknecht J. A. van der Gugten J. Hijzen T. H. Maes R. A. Hen R. Olivier B. (2001). Male and female 5-HT(1B) receptor knockout mice have higher body weights than wildtypes. *Physiol. Behav.* 74 507–516. 10.1016/s0031-9384(01)00589-3 11790410

[B15] Brivio P. Sbrini G. Peeva P. Todiras M. Bader M. Alenina N. (2018). TPH2 deficiency influences neuroplastic mechanisms and alters the response to an acute stress in a sex specific manner. *Front. Mol. Neurosci.* 11:389. 10.3389/fnmol.2018.00389 30425618 PMC6218558

[B16] Butovsky O. Ziv Y. Schwartz A. Landa G. Talpalar A. E. Pluchino S. (2006). Microglia activated by IL-4 or IFN-gamma differentially induce neurogenesis and oligodendrogenesis from adult stem/progenitor cells. *Mol. Cellul. Neurosci.* 31 149–160. 10.1016/j.mcn.2005.10.006 16297637

[B17] Chen J. Kang Q. Jiang W. Fan J. Zhang M. Yu S. (2015). The 5-HTTLPR confers susceptibility to anorexia nervosa in han chinese: evidence from a case-control and family-based study. *PLoS One* 10:e0119378. 10.1371/journal.pone.0119378 25785698 PMC4364880

[B18] Cisternas P. Salazar P. Serrano F. G. Montecinos-Oliva C. Arredondo S. B. Varela-Nallar L. (2015). Fructose consumption reduces hippocampal synaptic plasticity underlying cognitive performance. *Biochim. Biophys. Acta* 1852 2379–2390. 10.1016/j.bbadis.2015.08.016 26300486 PMC5369608

[B19] Commons K. G. (2009). Locally collateralizing glutamate neurons in the dorsal raphe nucleus responsive to substance P contain vesicular glutamate transporter 3 (VGLUT3). *J. Chem. Neuroanat.* 38 273–281. 10.1016/j.jchemneu.2009.05.005 19467322 PMC2767471

[B20] Conductier G. Crosson C. Hen R. Bockaert J. Compan V. (2005). 3,4- N -Methlenedioxymethamphetamine-induced hypophagia is maintained in 5-HT 1B receptor knockout mice, but suppressed by the 5-HT 2C receptor antagonist RS102221. *Neuropsychopharmacology* 30 1056–1063. 10.1038/sj.npp.1300662 15668722

[B21] Cowen P. J. Sargent P. A. Williams C. Goodall E. M. Orlikov A. B. (1995). Hypophagic, endocrine and subjective responses to m-chlorophenylpiperazine in healthy men and women. *Hum. Psychopharmacol. Clin. Exp.* 10 385–391. 10.1002/hup.470100504

[B22] Crabbe J. C. Metten P. Rhodes J. S. Yu C.-H. Brown L. L. Phillips T. J. (2009). A line of mice selected for high blood ethanol concentrations shows drinking in the dark to intoxication. *Biol. Psychiatry* 65 662–670. 10.1016/j.biopsych.2008.11.002 19095222 PMC3330756

[B23] Crabbe J. C. Spence S. E. Brown L. L. Metten P. (2011). Alcohol preference drinking in a mouse line selectively bred for high drinking in the dark. *Alcohol* 45 427–440. 10.1016/j.alcohol.2010.12.001 21194877 PMC3395368

[B24] Cui J. Yang K. Yu X. Wang J. Li J. Zhang Y. (2016). Chronic fluoxetine treatment upregulates the activity of the ERK1/2-NF-κB signaling pathway in the hippocampus and prefrontal cortex of rats exposed to forced-swimming stress. *Med. Princ. Pract.* 25 539–547. 10.1159/000449165 27532271 PMC5588511

[B25] Deacon R. M. J. (2006). Digging and marble burying in mice: simple methods for in vivo identification of biological impacts. *Nat. Protoc.* 1 122–124. 10.1038/nprot.2006.20 17406223

[B26] Demos K. E. Kelley W. M. Heatherton T. F. (2011). Dietary restraint violations influence reward responses in nucleus accumbens and amygdala. *J. Cogn. Neurosci.* 23 1952–1963. 10.1162/jocn.2010.21568 20807052 PMC3034108

[B27] Diaz S. L. Doly S. Narboux-Nême N. Fernández S. Mazot P. Banas S. M. (2012). 5-HT(2B) receptors are required for serotonin-selective antidepressant actions. *Mol. Psychiatry* 17 154–163. 10.1038/mp.2011.159 22158014 PMC3381222

[B28] Fuemmeler B. F. Agurs-Collins T. D. McClernon F. J. Kollins S. H. Kail M. E. Bergen A. W. (2008). Genes implicated in serotonergic and dopaminergic functioning predict BMI categories. *Obesity* 16 348–355. 10.1038/oby.2007.65 18239643 PMC2919156

[B29] Gould E. (1999). Serotonin and hippocampal neurogenesis. *Neuro-psychopharmacology* 21 46–51. 10.1016/S0893-133X(99)00045-7 10432488

[B30] Gras C. Herzog E. Bellenchi G. C. Bernard V. Ravassard P. Pohl M. (2002). A third vesicular glutamate transporter expressed by cholinergic and serotoninergic neurons. *J. Neurosci.* 22 5442–5451. 10.1523/JNEUROSCI.22-13-05442.2002 12097496 PMC6758212

[B31] Hare T. A. Camerer C. F. Rangel A. (2009). Self-control in decision-making involves modulation of the vmPFC valuation system. *Science (New York, N.Y.)* 324 646–648. 10.1126/science.1168450 19407204

[B32] Heisler L. K. Cowley M. A. Tecott L. H. Fan W. Low M. J. Smart J. L. (2002). Activation of central melanocortin pathways by fenfluramine. *Science (New York, N.Y.)* 297 609–611. 10.1126/science.1072327 12142539

[B33] Heisler L. K. Jobst E. E. Sutton G. M. Zhou L. Borok E. Thornton-Jones Z. (2006). Serotonin reciprocally regulates melanocortin neurons to modulate food intake. *Neuron* 51 239–249. 10.1016/j.neuron.2006.06.004 16846858

[B34] Herzog E. Gilchrist J. Gras C. Muzerelle A. Ravassard P. Giros B. (2004). Localization of VGLUT3, the vesicular glutamate transporter type 3, in the rat brain. *Neuroscience* 123 983–1002. 10.1016/j.neuroscience.2003.10.039 14751290

[B35] Higgs S. Robinson E. Lee M. (2012). Learning and memory processes and their role in eating: implications for limiting food intake in overeaters. *Curr. Obesity Rep.* 1 91–98. 10.1007/s13679-012-0008-9

[B36] Hioki H. Nakamura H. Ma Y.-F. Konno M. Hayakawa T. Nakamura K. C. (2010). Vesicular glutamate transporter 3-expressing nonserotonergic projection neurons constitute a subregion in the rat midbrain raphe nuclei. *J. Comp. Neurol.* 518 668–686. 10.1002/cne.22237 20034056

[B37] Jacques A. Chaaya N. Beecher K. Ali S. A. Belmer A. Bartlett S. (2019a). The impact of sugar consumption on stress driven, emotional and addictive behaviors. *Neurosci. Biobehav. Rev.* 103 178–199. 10.1016/j.neubiorev.2019.05.021 31125634

[B38] Jacques A. Chaaya N. Hettiarachchi C. Carmody M.-L. Beecher K. Belmer A. (2019b). Microtopography of fear memory consolidation and extinction retrieval within prefrontal cortex and amygdala. *Psychopharmacology* 236 383–397. 10.1007/s00213-018-5068-4 30610350

[B39] Jean A. Conductier G. Manrique C. Bouras C. Berta P. Hen R. (2007). Anorexia induced by activation of serotonin 5-HT4 receptors is mediated by increases in CART in the nucleus accumbens. *Proc. Natl. Acad. Sci. U.S.A.* 104 16335–16340. 10.1073/pnas.0701471104 17913892 PMC2042207

[B40] Jurdak N. Kanarek R. B. (2009). Sucrose-induced obesity impairs novel object recognition learning in young rats. *Physiol. Behav.* 96 1–5. 10.1016/j.physbeh.2008.07.023 18718844

[B41] Jurdak N. Lichtenstein A. H. Kanarek R. B. (2008). Diet-induced obesity and spatial cognition in young male rats. *Nutr. Neurosci.* 11 48–54. 10.1179/147683008X301333 18510803

[B42] Kanoski S. E. Davidson T. L. (2011). Western diet consumption and cognitive impairment: links to hippocampal dysfunction and obesity. *Physiol. Behav.* 103 59–68. 10.1016/j.physbeh.2010.12.003 21167850 PMC3056912

[B43] Kim S. Shou J. Abera S. Ziff E. B. (2018). Sucrose withdrawal induces depression and anxiety-like behavior by Kir2.1 upregulation in the nucleus accumbens. *Neuropharmacology* 130 10–17. 10.1016/j.neuropharm.2017.11.041 29191750

[B44] Kolodziejczak M. Béchade C. Gervasi N. Irinopoulou T. Banas S. M. Cordier C. (2015). Serotonin modulates developmental microglia via 5-HT2B receptors: potential implication during synaptic refinement of retinogeniculate projections. *ACS Chem. Neurosci.* 6 1219–1230. 10.1021/cn5003489 25857335

[B45] Kreisel T. Wolf B. Keshet E. Licht T. (2019). Unique role for dentate gyrus microglia in neuroblast survival and in VEGF-induced activation. *Glia* 67 594–618. 10.1002/glia.23505 30453385

[B46] Liu Z. Zhou J. Li Y. Hu F. Lu Y. Ma M. (2014). Dorsal raphe neurons signal reward through 5-HT and glutamate. *Neuron* 81 1360–1374. 10.1016/j.neuron.2014.02.010 24656254 PMC4411946

[B47] Mandyam C. D. Harburg G. C. Eisch A. J. (2007). Determination of key aspects of precursor cell proliferation, cell cycle length and kinetics in the adult mouse subgranular zone. *Neuroscience* 146 108–122. 10.1016/j.neuroscience.2006.12.064 17307295 PMC2230096

[B48] Maniam J. Antoniadis C. P. Youngson N. A. Sinha J. K. Morris M. J. (2016). Sugar consumption produces effects similar to early life stress exposure on hippocampal markers of neurogenesis and stress response. *Front. Mol. Neurosci.* 8:86. 10.3389/fnmol.2015.00086 26834554 PMC4717325

[B49] Mansouri-Guilani N. Bernard V. Vigneault E. Vialou V. Daumas S. Mestikawy S. E. (2019). VGLUT3 gates psychomotor effects induced by amphetamine. *J. Neurochem.* 148 779–795. 10.1111/jnc.14644 30556914

[B50] Mathes C. M. Gregson J. R. Spector A. C. (2013). The selective serotonin reuptake inhibitor paroxetine decreases breakpoint of rats engaging in a progressive ratio licking task for sucrose and quinine solutions. *Chem. Senses* 38 211–220. 10.1093/chemse/bjs096 23254343 PMC3569624

[B51] McDevitt R. A. Tiran-Cappello A. Shen H. Balderas I. Britt J. P. Marino R. A. (2014). Serotonergic versus non-serotonergic dorsal raphe projection neurons: differential participation in reward circuitry. *Cell Rep.* 8 1857–1869. 10.1016/j.celrep.2014.08.037 25242321 PMC4181379

[B52] Michael D. Martin K. C. Seger R. Ning M.-M. Baston R. Kandel E. R. (1998). Repeated pulses of serotonin required for long-term facilitation activate mitogen-activated protein kinase in sensory neurons of Aplysia. *Proc. Natl. Acad. Sci. U.S.A.* 95 1864–1869. 10.1073/pnas.95.4.1864 9465108 PMC19204

[B53] Miller B. R. Hen R. (2015). The current state of the neurogenic theory of depression and anxiety. *Curr. Opin. Neurobiol.* 30 51–58. 10.1016/j.conb.2014.08.012 25240202 PMC4293252

[B54] Miranda R. C. K. Genro J. P. Campagnolo P. D. B. Mattevi V. S. Vitolo M. R. Almeida S. (2017). Biallelic and triallelic approaches of 5-HTTLPR polymorphism are associated with food intake and nutritional status in childhood. *J. Nutr. Biochem.* 43 47–52. 10.1016/j.jnutbio.2017.01.015 28242565

[B55] National Health and Medical Research Council (2014). *Annual Report 2013-2014*. Canberra, ACT: National Health and Medical Research Council

[B56] Nonogaki K. Nozue K. Takahashi Y. Yamashita N. Hiraoka S. Kumano H. (2007). Fluvoxamine, a selective serotonin reuptake inhibitor, and 5-HT2C receptor inactivation induce appetite-suppressing effects in mice via 5-HT1B receptors. *Int. J. Neuropsychopharmacol.* 10 675–681. 10.1017/S1461145706007206 16959056

[B57] Papadeas S. T. Halloran C. McCown T. J. Breese G. R. Blake B. L. (2008). Changes in apical dendritic structure correlate with sustained ERK1/2 phosphorylation in medial prefrontal cortex of a rat model of dopamine D1 receptor agonist sensitization. *J. Comp. Neurol.* 511 271–285. 10.1002/cne.21835 18785628 PMC2587500

[B58] Patkar O. L. Belmer A. Beecher K. Jacques A. Bartlett S. E. (2019). Pindolol rescues anxiety-like behavior and neurogenic maladaptations of long-term binge alcohol intake in mice. *Front. Behav. Neurosci.* 13:264. 10.3389/fnbeh.2019.00264 31849624 PMC6895681

[B59] Pawluski J. L. Paravatou R. Even A. Cobraiville G. Fillet M. Kokras N. (2020). Effect of sertraline on central serotonin and hippocampal plasticity in pregnant and non-pregnant rats. *Neuropharmacology* 166:107950. 10.1016/j.neuropharm.2020.107950 31935392

[B60] Poirel O. Mamer L. E. Herman M. A. Arnulf-Kempcke M. Kervern M. Potier B. (2020). LSP5-2157 a new inhibitor of vesicular glutamate transporters. *Neuropharmacology* 164:107902. 10.1016/j.neuropharm.2019.107902 31811873

[B61] Qi J. Zhang S. Wang H.-L. Wang H. de Jesus Aceves Buendia J. Hoffman A. F. (2014). A glutamatergic reward input from the dorsal raphe to ventral tegmental area dopamine neurons. *Nat. Commun.* 5:5390. 10.1038/ncomms6390 25388237 PMC4231541

[B62] Reichelt A. C. (2016). Adolescent maturational transitions in the prefrontal cortex and dopamine signaling as a risk factor for the development of obesity and high fat/high sugar diet induced cognitive deficits. *Front. Behav. Neurosci.* 10:189. 10.3389/fnbeh.2016.00189 27790098 PMC5061823

[B63] Rhodes J. S. Best K. Belknap J. K. Finn D. A. Crabbe J. C. (2005). Evaluation of a simple model of ethanol drinking to intoxication in C57BL/6J mice. *Physiol. Behav.* 84 53–63. 10.1016/j.physbeh.2004.10.007 15642607

[B64] Rivera P. Hanamsagar R. Kan M. J. Tran P. K. Stewart D. Jo Y. C. (2019). Removal of microglial-specific MyD88 signaling alters dentate gyrus doublecortin and enhances opioid addiction-like behaviors. *Brain Behav. Immun.* 76 104–115. 10.1016/j.bbi.2018.11.010 30447281 PMC6348129

[B65] Rivera P. Pérez-Martín M. Pavón F. J. Serrano A. Crespillo A. Cifuentes M. (2013). Pharmacological administration of the isoflavone daidzein enhances cell proliferation and reduces high fat diet-induced apoptosis and gliosis in the rat hippocampus. *PLoS One* 8:e64750. 10.1371/journal.pone.0064750 23741384 PMC3669353

[B66] Roepke T. A. Smith A. W. Rønnekleiv O. K. Kelly M. J. (2012). Serotonin 5-HT2C receptor-mediated inhibition of the M-current in hypothalamic POMC neurons. *Am. J. Physiol. Endocrinol. Metab.* 302 E1399–E1406. 10.1152/ajpendo.00565.2011 22436698 PMC3378066

[B67] Sakae D. Y. Ramet L. Henrion A. Poirel O. Jamain S. El Mestikawy S. (2019). Differential expression of VGLUT3 in laboratory mouse strains: impact on drug-induced hyperlocomotion and anxiety-related behaviors. *Genes Brain Behav.* 18:e12528. 10.1111/gbb.12528 30324647

[B68] Sargent P. A. Sharpley A. L. Williams C. Goodall E. M. Cowen P. J. (1997). 5-HT2C receptor activation decreases appetite and body weight in obese subjects. *Psychopharmacology* 133 309–312. 10.1007/s002130050407 9361339

[B69] Sengupta A. Bocchio M. Bannerman D. M. Sharp T. Capogna M. (2017). Control of amygdala circuits by 5-HT neurons via 5-HT and glutamate cotransmission. *J. Neurosci.* 37 1785–1796. 10.1523/JNEUROSCI.2238-16.2016 28087766 PMC5320609

[B70] Shariff M. Quik M. Holgate J. Morgan M. Patkar O. L. Tam V. (2016). Neuronal nicotinic acetylcholine receptor modulators reduce sugar intake. *PLoS One* 11:e0150270. 10.1371/journal.pone.0150270 27028298 PMC4814119

[B71] Sierra A. Encinas J. M. Deudero J. J. P. Chancey J. H. Enikolopov G. Overstreet-Wadiche L. S. (2010). Microglia shape adult hippocampal neurogenesis through apoptosis-coupled phagocytosis. *Cell Stem Cell* 7 483–495. 10.1016/j.stem.2010.08.014 20887954 PMC4008496

[B72] Smith S. R. Prosser W. A. Donahue D. J. Morgan M. E. Anderson C. M. Shanahan W. R. (2009). Lorcaserin (APD356), a selective 5-HT(2C) agonist, reduces body weight in obese men and women. *Obesity (Silver Spring, Md.)* 17 494–503. 10.1038/oby.2008.537 19057523

[B73] Sohn J.-W. Xu Y. Jones J. E. Wickman K. Williams K. W. Elmquist J. K. (2011). Serotonin 2C receptor activates a distinct population of arcuate pro-opiomelanocortin neurons via TRPC channels. *Neuron* 71 488–497. 10.1016/j.neuron.2011.06.012 21835345 PMC3184528

[B74] Sookoian S. Gemma C. García S. I. Gianotti T. F. Dieuzeide G. Roussos A. (2007). Short Allele of Serotonin Transporter gene promoter is a risk factor for obesity in adolescents. *Obesity* 15 271–276. 10.1038/oby.2007.519 17299098

[B75] Tecott L. H. Sun L. M. Akana S. F. Strack A. M. Lowenstein D. H. Dallman M. F. (1995). Eating disorder and epilepsy in mice lacking 5-HT2c serotonin receptors. *Nature* 374 542–546. 10.1038/374542a0 7700379

[B76] Thiele T. E. Crabbe J. C. Boehm S. L. (2014). “Drinking in the Dark” (DID): a simple mouse model of binge-like alcohol intake. *Curr. Protoc. Neurosci.* 68 9.49.1–9.49.12. 10.1002/0471142301.ns0949s68 24984686 PMC4142649

[B77] Tukey D. S. Lee M. Xu D. Eberle S. E. Goffer Y. Manders T. R. (2013). Differential effects of natural rewards and pain on vesicular glutamate transporter expression in the nucleus accumbens. *Mol. Brain* 6:32. 10.1186/1756-6606-6-32 23835161 PMC3710235

[B78] van der Borght K. Köhnke R. Göransson N. Deierborg T. Brundin P. Erlanson-Albertsson C. (2011). Reduced neurogenesis in the rat hippocampus following high fructose consumption. *Regul. Pept.* 167 26–30. 10.1016/j.regpep.2010.11.002 21115071

[B79] Vrettou M. Nilsson K. W. Tuvblad C. Rehn M. Åslund C. Andershed A.-K. (2019). VGLUT2 rs2290045 genotype moderates environmental sensitivity to alcohol-related problems in three samples of youths. *Eur. Child Adolesc. Psychiatry* 28 1329–1340. 10.1007/s00787-019-01293-w 30805764 PMC6785645

[B80] Walf A. A. Frye C. A. (2007). The use of the elevated plus maze as an assay of anxiety-related behavior in rodents. *Nat. Protoc.* 2 322–328. 10.1038/nprot.2007.44 17406592 PMC3623971

[B81] Wallace J. Lord J. Dissing-Olesen L. Stevens B. Murthy V. N. (2020). Microglial depletion disrupts normal functional development of adult-born neurons in the olfactory bulb. *ELife* 9:e50531. 10.7554/eLife.50531 32150529 PMC7062469

[B82] Walsh A. E. Smith K. A. Oldman A. D. Williams C. Goodall E. M. Cowen P. J. (1994). M-Chlorophenylpiperazine decreases food intake in a test meal. *Psychopharmacology* 116 120–122. 10.1007/BF02244883 7862925

[B83] Wang H.-L. Zhang S. Qi J. Wang H. Cachope R. Mejias-Aponte C. A. (2019). Dorsal raphe dual serotonin-glutamate neurons drive reward by establishing excitatory synapses on VTA mesoaccumbens dopamine neurons. *Cell Rep.* 26 1128–1142.e7. 10.1016/j.celrep.2019.01.014 30699344 PMC6489450

[B84] Xu T. J. Reichelt A. C. (2018). Sucrose or sucrose and caffeine differentially impact memory and anxiety-like behaviours, and alter hippocampal parvalbumin and doublecortin. *Neuropharmacology* 137 24–32. 10.1016/j.neuropharm.2018.04.012 29729502

[B85] Xu Y. Berglund E. D. Sohn J.-W. Holland W. L. Chuang J.-C. Fukuda M. (2010). 5-HT2CRs expressed by Pro-opiomelanocortin neurons regulate insulin sensitivity in liver. *Nat. Neurosci.* 13 1457–1459. 10.1038/nn.2664 21037584 PMC3059249

[B86] Xu Y. Jones J. E. Kohno D. Williams K. W. Lee C. E. Choi M. J. (2008). 5-HT2CRs expressed by pro-opiomelanocortin neurons regulate energy homeostasis. *Neuron* 60 582–589. 10.1016/j.neuron.2008.09.033 19038216 PMC2631191

[B87] Yankelevitch-Yahav R. Franko M. Huly A. Doron R. (2015). The forced swim test as a model of depressive-like behavior. *J. Vis. Exp.* 97:52587. 10.3791/52587 PMC4401172 25867960

[B88] Yoo D. Y. Kim W. Kim D. W. Nam S. M. Jung H. Y. Kim J. W. (2014). Cell proliferation and neuroblast differentiation in the dentate gyrus of high-fat diet-fed mice are increased after rosiglitazone treatment. *J. Vet. Sci.* 15 27–33. 10.4142/jvs.2014.15.1.27 24136217 PMC3973763

[B89] Yuen E. Y. Jiang Q. Chen P. Feng J. Yan Z. (2008). Activation of 5-HT2A/C receptors counteracts 5-HT1A regulation of N-Methyl-D-aspartate receptor channels in pyramidal neurons of prefrontal cortex. *J. Biol. Chem.* 283 17194–17204. 10.1074/jbc.M801713200 18442977 PMC2427346

[B90] Zou W.-J. Song Y.-L. Wu M.-Y. Chen X.-T. You Q.-L. Yang Q. (2020). A discrete serotonergic circuit regulates vulnerability to social stress. *Nat. Commun.* 11:4218. 10.1038/s41467-020-18010-w 32839452 PMC7445164

