# Supplementary figures and images for: Sucrose Consumption Alters Serotonin/Glutamate Co-localisation Within the Prefrontal Cortex and Hippocampus of Mice
Source: Front Mol Neurosci. 2021 Jun 28;14:678267. doi: 10.3389/fnmol.2021.678267 (PMC8273284; doi:10.3389/fnmol.2021.678267)

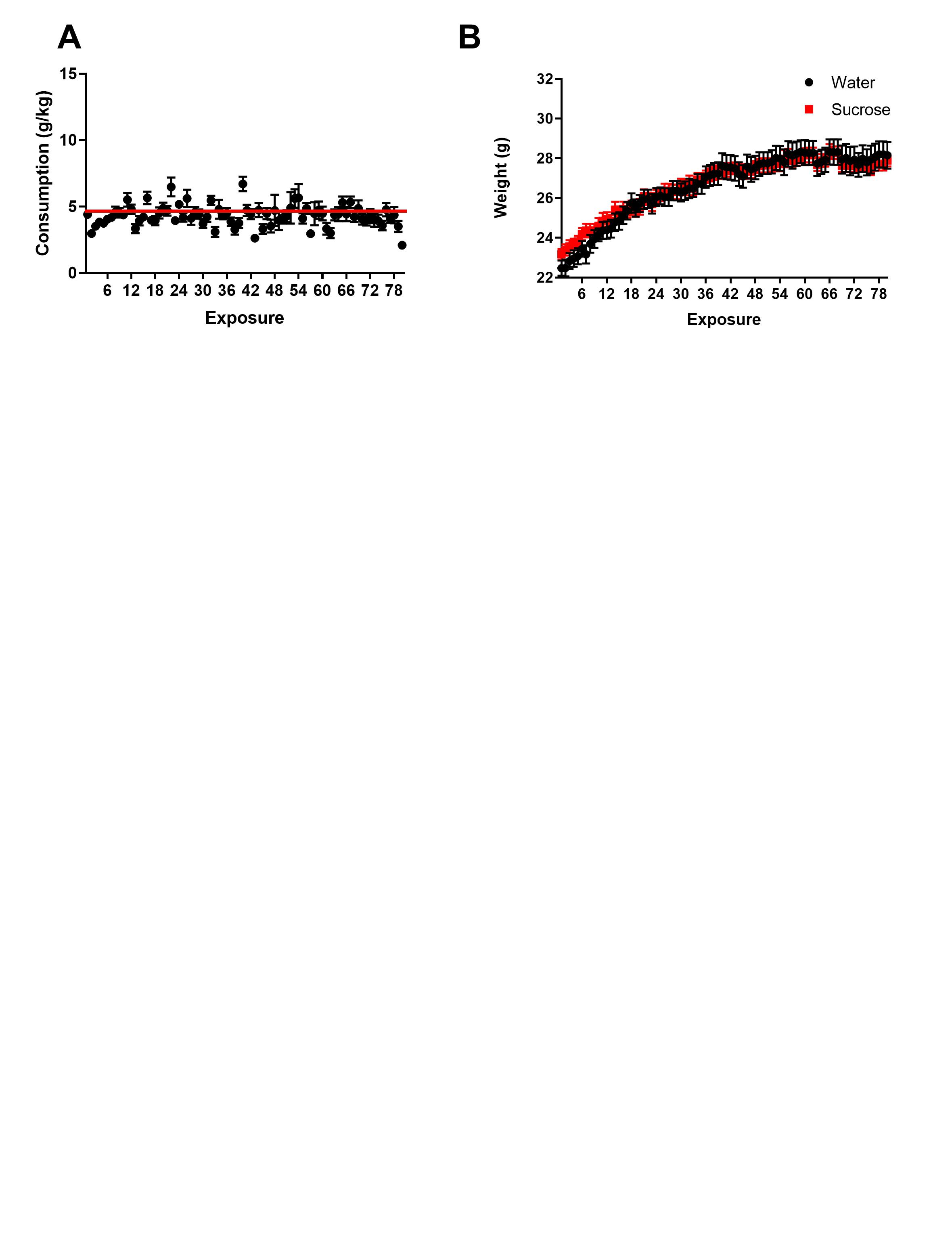

Supplement: Supplementary Figure 1 — Five percentage sucrose consumption and weight. (A) After 16 weeks of access to 25% sucrose, mice exhibited stable levels of sucrose intake around 3–6 g/kg/2 h (mean 4.4 g/kg/2 h indicated by the red line). (B) No weight change was observed across the 16 weeks. Data are presented as mean ± S.E.M; n = 8 mice/group. [file Image_1.JPEG]

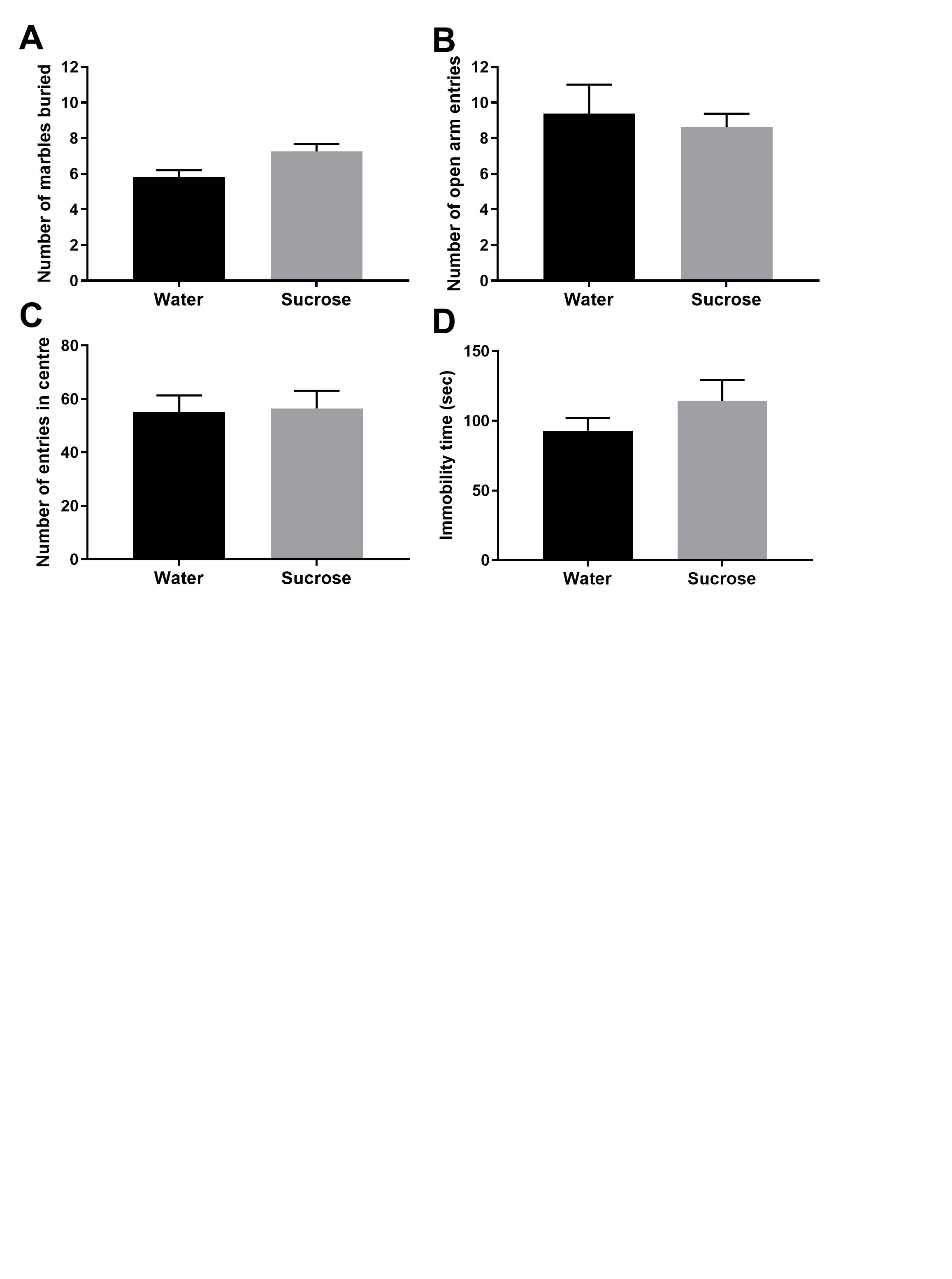

Supplement: Supplementary Figure 2 — No change observed in anxious behaviour as seen by the number of marbles buried in marble burying test (A), number of open arm entries in the elevated-plus-maze (B), number of entries into the centre in the open-field test (C), no change in depressive like behaviour as seen by immobility time in the forced swimming test (D). Data are presented as mean ± S.E.M (t-test); n = 8 mice/group. [file Image_2.JPEG]

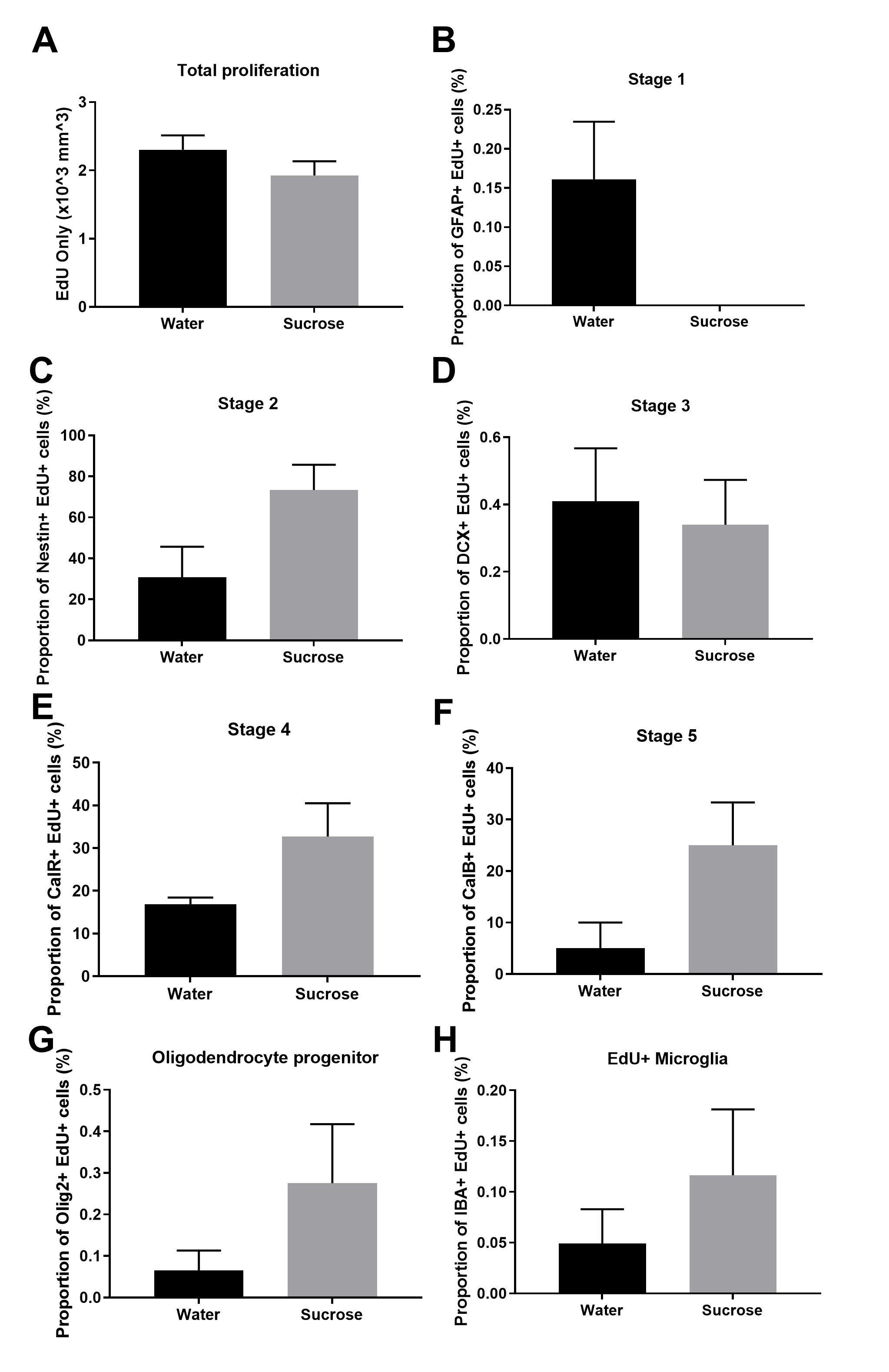

Supplement: Supplementary Figure 3 — (A) No change in the total number of EdU + cells, suggesting no change in overall proliferation. No change in proportion of EdU + co-localised with any stage of neurogenesis: stage 1: glial fibrillary acidic protein (GFAP, B); stage 2: Nestin (C); stage 3: Doublecortin (DCX, D); stage 4: calretinin (CalR, E) and stage 5: calbindin (CalB, F) suggesting no change in neurogenesis. No change in proportion of EdU + co-localised with glial populations: oligodendrocyte (olig2, G) and microglia (IBA, H). Data are presented as mean ± S.E.M (t-test); n = 8 mice/group. [file Image_3.JPEG]
